# Trounecek Serum

**Published:** 1913-02

**Authors:** 


Trounecek Serum.—The *American Practitioner* recurs to this method for Arteriosclerosis. The formula is:

| | | |
|---|---|---|
| Sodium chlorid .................... | 10. | grammes |
| Sodium sulphate .................... | 1. | " |
| Calcium phosphate .................. | .75 | " |
| Magnesium phosphate ............... | .75 | " |
| Sodium carbonate ................... | .40 | " |
| Sodium phosphate ................... | .30 | " |

1 gramme of the mixture is dissolved in 15 c. c. of sterile water. 2 c. c. is injected into the buttocks alternate days, increasing the dose by 1 c. c. till 8 c. c. are given. Obviously, the solution of the powder is best accomplished by making stock solutions and these will show a precipitate as is obvious from the composition. Personally, we cannot see the sense of using calcium in arteriosclerosis nor of introducing carbonates. We have, therefore, followed the formula of blood plasma, omitting carbonates which are adventitiously due to the presence of carbon dioxid, as well as lime, and distributing the acid radicles among the three other metallic bases. Chemically pure salts must be used, table salt containing appreciable quantities of lime which precipitate with sulphates. While theoretically advisable, we must confess never to have seen any conspicuous advantage from this method and have simplified it in most cases to prescribing chemically pure salt for use in the kitchen and on the table.

